# Nomogram predicts survival benefit from preoperative radiotherapy for non-metastatic breast cancer: A SEER-based study

**DOI:** 10.18632/oncotarget.17991

**Published:** 2017-05-18

**Authors:** Jianjun Liu, Mingxue Su, Shikai Hong, Hong Gao, Xucai Zheng, Shengying Wang

**Affiliations:** ^1^ Department of Head, Neck, and Breast Surgery, Anhui Provincial Cancer Hospital, West Branch of Anhui Provincial Hospital, Hefei, China; ^2^ Department of Infectious Disease Epidemiology, Lu'an People's Hospital, Lu'an, China

**Keywords:** nomogram, breast cancer, preoperative radiotherapy

## Abstract

**Background:**

To estimate survival in non-metastatic breast cancer patients who failed to achieve a pathological complete response (pCR) more effectively, we combined the clinicpathological characteristics after preoperative radiation therapy (pRT) and established a novel nomogram.

**Materials and Methods:**

Using the Surveillance, Epidemiology, and End Results (SEER) database, we identified 2,545 non-metastatic breast cancer patients who underwent pRT between 1998 and 2013. Based on the registries of patients, the primary cohort divided into training set (*n* = 1,692) and validation set (*n* = 853). Nomograms were established by training set and validated by validation set.

**Results:**

According to the multivariate analysis of training set, nomogram which combined age at diagnosed, marital status, location, grade, ER status, yp-T status, yp-N status and whether received breast conservation surgery (BCS) was developed. Calibration plots of the nomograms showed that the probability of DSS corresponded to actual observation closely. The C-index was 0.78 in validation set, which was significantly higher than that of yp-TNM staging system (0.75, *p* = 0.004).

**Conclusions:**

The proposed nomogram resulted in more–reliable DSS prediction for non-metastatic breast cancer patients in general population, it would be helpful in individualized survival prediction and better treatment allocation after pRT.

## INTRODUCTION

Breast cancer is one of the most frequently diagnosed cancer in Western country. In 2016, it was estimated that more than 249,000 new breast cancer patients was diagnosed, and it accounted for nearly 30% of all incident cancer in females. In addition, the breast cancer is an aggressive cancer with poor survival, especially in the patients with locally advanced stage or distant metastasis [1]. Neoadjuvant therapy (NAT) may be the only potential curative treatment for locally advanced, non-distant metastatic breast cancer patients [2, 3]. Furthermore, several studies have demonstrated that NAT can induce tumor-downstaging, thereby promote breast conservation surgery rate for non-metastatic breast cancer patients [4, 5].

Moreover, its can reduce tumor burden, as well as provide a unique opportunity to estimate the outcome of patients by the postoperative pathology [6]. The findings of numerous previous studies have demonstrated that the pCR rate was a strong and acceptable predictor for breast cancer [6–8]. Based on the status of response for NAT, the patients can be divided into two groups, pCR group and non-pCR group. However, there were only few patients that could achieve pCR, more than 80% patients were diagnosed as tumor residual [9]. In fact, this classification oversimplified the different prognostic categories for the breast cancer patients, especially for the patients with tumor residual.

Currently, the tumor residual burden is evaluated by AJCC stage system, this staging system assumes that tumor cells spread sequentially, which means tumor cells spread firstly from the primary site to lymphatic system, and then translate to distant organs. [10] This strategy solely depends on the final pathologic stage for stratifying patients. However, due to the tumor-downstaging of NAT, this classification may not be applicable for the patients after NAT. In addition, patients' survival was also effected by other individual factors. It is believed that host status and other prognostic factors such as age, race, histology and different treatments after surgery could significantly affect the individual survival in some tumors. [11–13]. Thus, a more accurate survival prediction model incorporating more individual factors was needed.

Recently, Nomogram, a simple predictive tool, have been constructed in several tumors and proved to be useful and effective. [14–18] However, the nomogram applicable for breast cancer after pRT has not been constructed. In this study, we aimed to develop and validate a nomogram based on a multi–institution and multi–population data from SEER database to estimate the survival of patients with non-metastatic breast cancer after pRT.

## RESULTS

### Clinicopathologic characteristics of patients and follow-up

In primary cohort, a total of 2,545 non-metastatic breast cancer patients who received pRT were included. The clinicopathologic characteristics of breast cancer patients form the training set (*n* = 1,692) and validation set (*n* = 853) were listed in Table 1, respectively. Overall, the mean age at diagnosed was 57.3 ± 13.3 years. The majority race of patients was white (84.0%). The majority surgery after radiotherapy was breast conservation surgery (65.9%). 59.7% patients with pathological lymph node-negative. Additionally, 32.0% patients received radiotherapy after surgery (ART). The median follow-up was 95.9 months. 848 (33.3%) patients died before the analysis of the present study. The 3-year, 5-year, 10-year DSS were 88.7%, 84.0% and 75.7%, respectively.

**Table 1:** Characteristic of training set and validation set

	Training set (*n* = 1,692)		Validation set (*n* = 853)	
Characteristic	NO.	%	NO.	%
Age (years)				
Mean	58.0 ± 13.3		55.9 ± 13.2	
Range	25 to 93		25 to 96	
Sex				
Male	5	0.3	3	0.4
Female	1687	99.7	850	99.6
Marital				
Yes	1250	73.9	621	72.8
No	442	26.1	232	27.2
Race				
White	1479	87.4	659	77.3
Black	157	9.3	119	14.0
API/AI	56	3.3	75	8.8
Tumor location				
Center/Nipple	92	5.4	71	8.3
Upper-outer	771	45.6	346	40.6
Upper-inner	200	11.8	91	10.7
Lower-outer	138	8.2	60	7.0
Lower-inner	112	6.6	57	6.7
Overlapping lesion	379	22.4	228	26.7
Grade				
Well	290	17.1	106	12.4
Moderately	768	45.4	315	36.9
Poorly	606	35.8	418	49.0
Undifferentiated	28	1.7	14	1.6
Yp-T stage				
1	1020	60.3	348	40.8
2	462	27.3	250	29.3
3	106	6.3	111	13.0
4	104	6.1	144	16.9
yp-N stage.				
0	1091	64.5	429	50.3
1	340	20.1	263	30.8
2	167	9.9	108	12.7
3	94	5.6	53	6.2
ER status				
Negative	408	24.1	289	33.9
Positive	1284	75.9	564	56.1
PR status				
Negative	587	34.7	389	45.6
Positive	1105	65.3	451	52.9
Unknown	1105	65.3	13	1.5
yp-AJCC Stage				
I	827	48.9	261	30.6
II	495	29.3	290	34.0
III	370	21.9	302	35.4
BCS				
Yes	1219	72.0	457	53.6
No	473	28.0	396	46.4
ART				
Yes	518	30.6	297	34.8
No	1174	69.4	556	65.2

Abbreviation: API, Asian or Pacific Islander; AI, Asian or Pacific Islander; ER status: Estrogen Receptor status; PR status: Progesterone Receptor status; BCS, Breast Conservation Surgery; ART, Adjuvant radiotherapy; AJCC, American Joint Committee on Cancer.

### Independent risk factors associated with survival in training set and nomogram development

According to Harrell's guidelines, Clinical pathological variables should been transformed and examined to fit the Cox PH regression and linear assumption before models construction [19]. The continuous variables were translated into category variables by X-tile software. The best cutoff points for age at diagnosed were 64 and 75 years old (Supplementary Figure 1). In the univariate analysis, most clinicopathologic characteristics, such as Age at diagnosed, marital status, race, grade, tumor location, yp-T stage, yp-N stage, ER status, PR status, BCS and ART, were associated with survival. All the significant factors were included into multivariate analysis.

**Figure 1:**
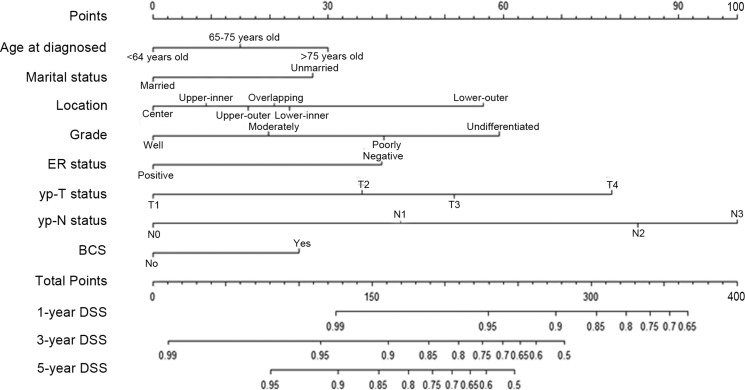
Nomogram predicting 1–year, 3–year and 5–year DSS for non-metastasis breast cancer patients after pRT The nomogram was used by accumulating the points identified on the points scale for each variable. Based the sum of these points projected on the bottom scales, the nomogram can provide the probability of 1–year, 3–year and 5–year DSS for an individual patient. Abbreviation: ER, estrogen receptor; DSS, disease specific survival; BCS, breast conservation surgery; pRT, neoadjuvant radiotherapy; API, Asian or Pacific Islander; AI, Asian or Pacific Islander.

The result of multivariate analysis was listed in Table 2. Based on the result of multivariate analysis, nomogram predicting 1-year, 3-year and 5-year DSS was constructed (Figure 1).

**Table 2:** Multivariate analysis of the training set

	HR	95% CI	*p*
Age (years)			0.017
< 64	ref		
65∼75	1.22	0.93 to 1.61	
> 76	1.62	1.14 to 2.30	
Race			
White			
Black			
API/AI			
Marital			< 0.001
Yes	ref		
No	0.65	0.52 to 0.83	
Location			0.018
Center/Nipple	ref		
Upper- inner	1.15	0.65 to 2.01	
Upper- outer	1.28	0.81 to 2.02	
Overlapping lesion	1.37	0.85 to 2.21	
Lower- inner	1.43	0.81 to 2.54	
Lower- outer	2.38	1.37 to 4.14	
Grade			0.007
Well	ref		
Moderately	1.44	0.92 to 2.27	
Poorly	1.90	1.20 to 3.020	
Undifferentiated	2.72	1.35 to 5.48	
yp-T stage			< 0.001
1	ref		
2	1.75	1.32 to 2.31	
3	2.23	1.45 to 3.44	
4	3.39	2.28 to 5.04	
yp-N stage			< 0.001
0	ref		
1	1.93	1.43 to 2.60	
2	3.62	2.59 to 5.05	
3	4.75	3.25 to 6.94	
ER status			< 0.001
Positive	ref		
Negative	0.54	0.43 to 0.69	
BCS			0.011
Yes	ref		
No	0.68	0.50 to 0.91	

Abbreviation: HR: Hazard Ratio; API, Asian or Pacific Islander; AI, Asian or Pacific Islander; ER status: Estrogen Receptor status; BCS, Breast Conservation Surgery.

### Nomogram validation

The nomogram was initially validated by bootstrap validation, and then Cross-validated by validation set. As the bootstrap validation result shown, the nomogram model demonstrated more-accuracy for predicting DSS, with an unadjusted C-index of 0.80 and a bootstrap-adjusted C-index of 0.79, which was higher than that of yp-TNM staging system (0.75). The result was similar to the cross-validation. The C-index of the DSS-model was 0.78 (95%CIs, 0.75–0.80), which was significant higher than yp-TNM staging system (0.75, 95%CIs, 0.72–0.78, *p* = 0.004).

**Figure 2:**
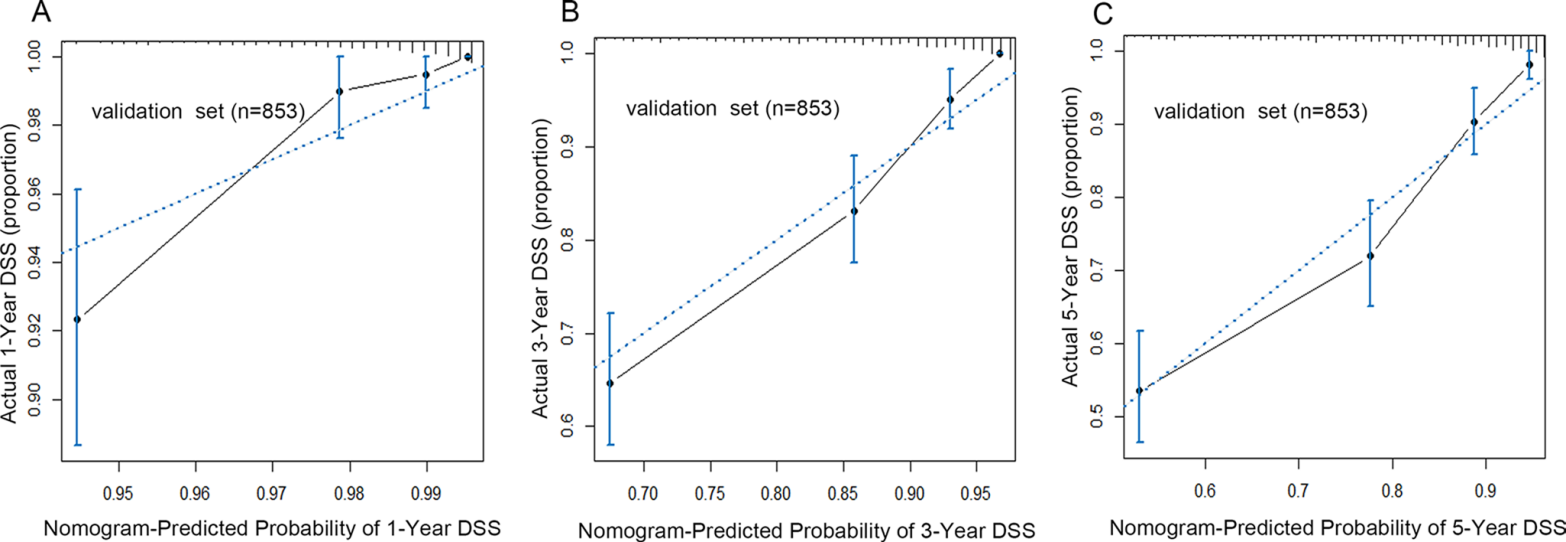
The calibration curve for predicting patients' DSS at 1–year (**A**), 3–year (**B**) and 5–year (**C**) in the validation set. The X–aixs represented the nomogram–predicted survival, and the actual survival was plotted on the Y–axis. The dotted line represented the ideal correlationship between nomogram-predicted and actual survival. Abbreviation: DSS, disease specific survival.

**Figure 3:**
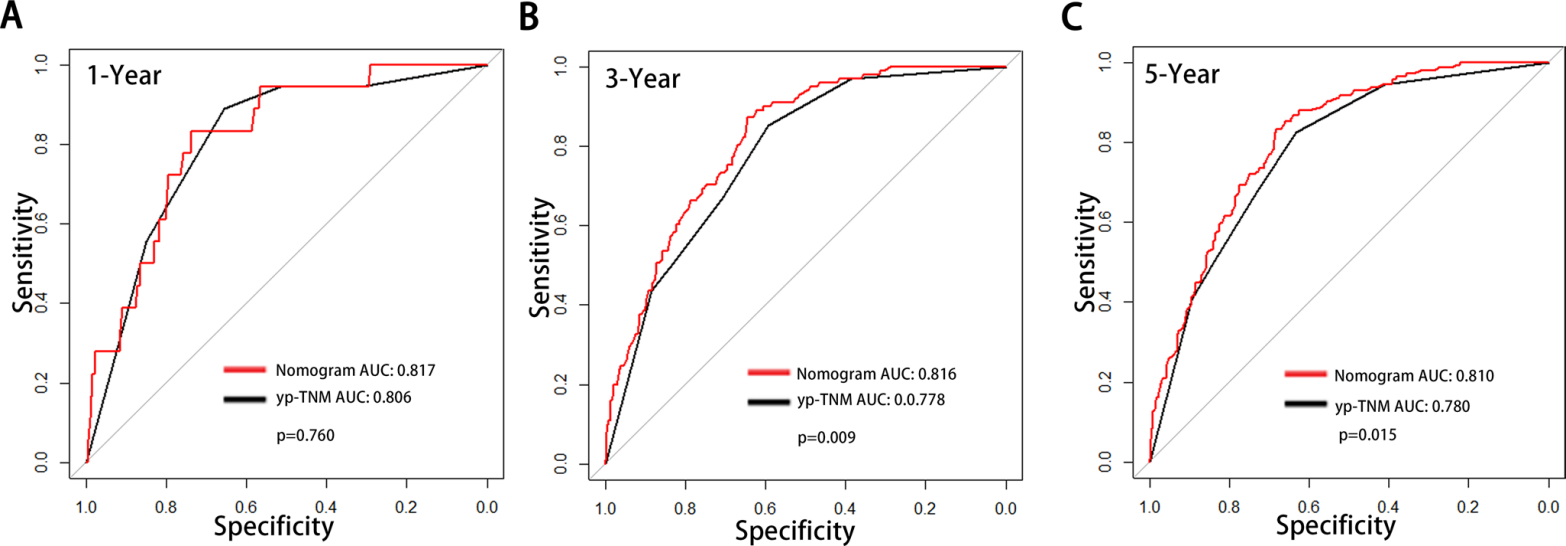
Comparison of the areas under the receiver operating curves of two prognostic models to prediction of DSS at 1–year (A), 3–year (B) and 5–year (C) in the validation set The red lines represent nomogram predicted DSS and the balack lines represent the yp-TNM staging predicted DSS. Abbreviation: DSS, Disease Specific Survival.

The calibration curves for 1-year, 3-year and 5-year DSS were performed in validation set, and the plots showed that DSS corresponded closely to the actual survival estimated by the Kaplan–Meier method in the validation set (Figure 2). In addition, by the AUC (area of ROC curve) analysis, we also compared the DSS predicting ability of the two models in each time points (Figure 3). As shown in the Figure 3, the proposed nomogram shows more accurate survival predictive ability than the yp-TNM staging system in 3-year and 5-year DSS predicting.

## DISCUSSION

In the present study, a total of 2,454 non-metastatic breast cancer patients undergoing pRT from SEER database were analyzed. Based on the clinicopathologic characteristics, we first developed and validated a nomogram to estimate the probability DSS, which exhibited more accuration survival prediction than the yp-TNM staging system.

At present, the models based on postoperative pathology characteristics to predict survival for breast cancer underwent preoperative therapy were still controversy. In 2008, Guarneri et al identified that lymph node status and Ki-67 were the only independent risk factors for breast cancer patients underwent neoadjuvant chemotherapy (NAC) [20]. Ki-67 expression more than 15%, and lymph node metastasis were associated with worse DSS (HR = 3.75, *P* < 0.001, HR = 2.31, *P* = 0.037, respectively). Based on those findings, the authors proposed a new classification including Ki-67 and lymph node status for breast cancer patients underwent NAC. Those findings were interesting, however, the sample size of that study was small. The selection bias may exist. Furthermore, several other important clinicopathologic characteristics had been ignored. For example, the tumor location, patients' age at diagnosed and the postoperative treatment, which may also affect patients' survival. It is believed that a more refined prognostic classification which included more risk factors for breast cancer patients was needed.

To date, several nomograms had been constructed, and shown more accurate survival prediction in breast cancer patients underwent NAC. In 2005, based on 1,070 breast cancer patients with pCR evaluation forming various clinical trials, Rouzier and colleagues developed and validated a nomogram, which included clinical stage, ER status, grade and the number of preoperative chemotherapy cycles to estimate distant metastasis-free survival [21]. This nomogram was validated by two independent institution data, and the C-index were both higher than 0.77. This nomogram was important, it was the first nomogram to predict breast cancer patients undergoing preoperative therapy. However, according to Harrell's guidelines, this nomogram was based on the pCR patients, it was not suitable for the patients with tumor residual. In 2011, Keam et al developed a nomogram, which included age at diagnosed, initial clinical stage, yp-TNM stage, ER status and Ki-67. Its C-index was 0.78, and could be used to predict the probability of 2-year relapse-free survival. However, the patients of this study were all received NAC, it might not be suitable for patients undergoing pRT.

In this study, we first analyzed a multi–institution and multi–population database, and identified the risk factors for breast cancer underwent pRT. Besides lymph node status, we identified several other clinicopathologic characteristics associated with patients' survival. Combining all the risk factors, we developed and validated DSS-model to predict breast cancer patients' survival, and the nomogram exhibited more-accuracy survival prediction than yp-TNM staging system. Indeed, it would be helpful to design an individual postoperative treatment and the schedule of follow-up for breast cancer patient.

Although the nomogram demonstrated a more-accuracy survival prediction, several limitations should not be ignored. Firstly, there may be a selection bias in the primary cohort, since only the patients who had complete information were included in present study. Secondly, as those nomograms were based on SEER database, all the analysis were limited to the prognosis factors in the database. Several predictors such as, Ki-67 index, tumor genetic differences, whether received neoadjuvant chemotherapy, hormone therapy and postoperative chemotherapies had not been analyzed. Lastly, patients included in the study may also had received other therapy, such as NAC and/or preoperative hormone therapy, which may limit the statistics power of this study.

In conclusion, our study first developed and validated a prognostic nomogram based on a multi–institution and multi–population database predicting DSS for breast cancer patients undergoing pRT. Compared to the yp-TNM staging system, the proposed nomogram represented better prognostic discrimination and predictive accuracy for DSS. It should be helpful to calculate individualized survival prediction and provide better treatment allocation for non-metastasis breast cancer after pRT.

## MATERIALS AND METHODS

### Patients

The primary cohort was derived from the SEER program. The SEER program is a national collaboration program by the National Cancer Institute of Unit State. Approximately 3 million cases from a variety of geographic regions had been collected and published, and it covers nearly 26% American population's cancer incidence and survival data.

A retrospective review of all breast cancer patients after pRT with tumor residual from SEER database between 1998 and 2013 was performed. A total of 7,310 patients from 18 SEER registries were initially screened. Since the patients with distant metastasis have an obviously worse survival than non-metastasis patients, those patients were not analyzed in current study. In addition, patients were excluded if they had incomplete information(s) on tumor size (yp-T stage), lymph node stage (yp-N stage) or status of distant organs metastasis (M stage). The remained (*n* = 2,545) were defined as SEER primary cohort. In the SEER primary cohort, patients from 7 randomly selected registries (Louisiana, New Jersey, New Mexico, Rural Georgia, San Francisco-Oakland SMSA, San Jose-Monterey, Seattle, Utah) were assigned as training data set, and from other registries were regarded as SEER validation set.

### Outcomes

The primary endpoint was DSS, which was defined as the time form surgery to cancer–related death or the last follow–up. The follow–up duration was measured as the time from the date of surgery to the last follow–up. The survival status was recorded according to the latest follow–up.

### Covariates

The data of patients' clinicpathological characteristics such as age at diagnosed, sex, race, marital, surgery, tumor location, tumor size (continuous variable), histology, grade, yp-T stage (category variable), yp-N stage, estrogen receptor (ER) status, progesterone receptor (PR) status, M stage, number of positive metastasis lymph nodes and number of examined lymph nodes were collected. The pathological tumor stage, yp-T stage and yp-N stage were restaged according to the 7th edition of AJCC staging system.

### Risk factors selected and the construction of the nomogram

The method was performed as our previous studies described [22, 23]. Briefly, the nomogram assumes that a linear correlation between risk factors and patients survival [19]. The linear relationships between continuous variables and survival were evaluated by restricted cubic splines. Before modeling, continuous variables were transformed into categorical variables to fit the linear assumption. The best cut-off points of continuous variables were identified by X-tile [24]. Categorical variables were grouped based on clinical findings before modeling. By the forward stepwise in the Cox proportional hazards (PH) regression model, all the independent risk factors were identified. DSS estimation and survival curve were performed by Kaplan–Meier method, and validated by the log–rank test.

The model of nomogram was established based on the data of training set. According to the results of Cox PH regression, nomogram combining all the independent prognostic factors was constructed for predicting 1–year, 3–year and 5–year DSS by using the package of *rms* in R software version 3.2.4.

### Validation of the nomogram

The nomogram was validated by measuring both discrimination and calibration in validation dataset. Firstly, Discrimination between the proposed nomogram and the 7th edition of AJCC staging system were performed by the *roccp.cens* package in R. Bootstrapping is an internal validation whereby the model is iteratively applied to randomly selected sample sets of the primary cohort. However, external validation is performed in many disparate cohorts, which is the gold standard validation. Therefore, in this part, the nomogram was internal validated by bootstrap in the training set and external vxalidated in the validation set. The discrimination of nomogram was evaluated by Harrell's C–index, which could estimate the probability between the observed and predicted DSS [19]. The higher the C–index, the more precise the survival prediction was. Following, calibrations were firstly carried out by grouping all the patients, and then the mean of the groups were compared with observed Kaplan–Meier DSS estimation.

### Statistical analysis

All the *p value* less than 0.05 will be considered as statistically significant. All statistics analysis were performed by the R software version 3.2.4 (http://www.r-project.org), X-title and the software statistical package for social sciences version 19.0 (SPSS, Chicago, IL).

## SUPPLEMENTARY FIGURE


