# Supplementary material for: Nomogram predicts survival benefit from preoperative radiotherapy for non-metastatic breast cancer: A SEER-based study
Source: Oncotarget. 2017 May 18;8(30):49861–8. doi: 10.18632/oncotarget.17991 (PMC5564813; doi:10.18632/oncotarget.17991)
Supplement: Supplementary file 1 [file oncotarget-08-49861-s001.pdf]

Nomogram predicts survival benefit from preoperative radiotherapy for non-metastatic breast cancer: A SEER-based study

Supplementary Materials

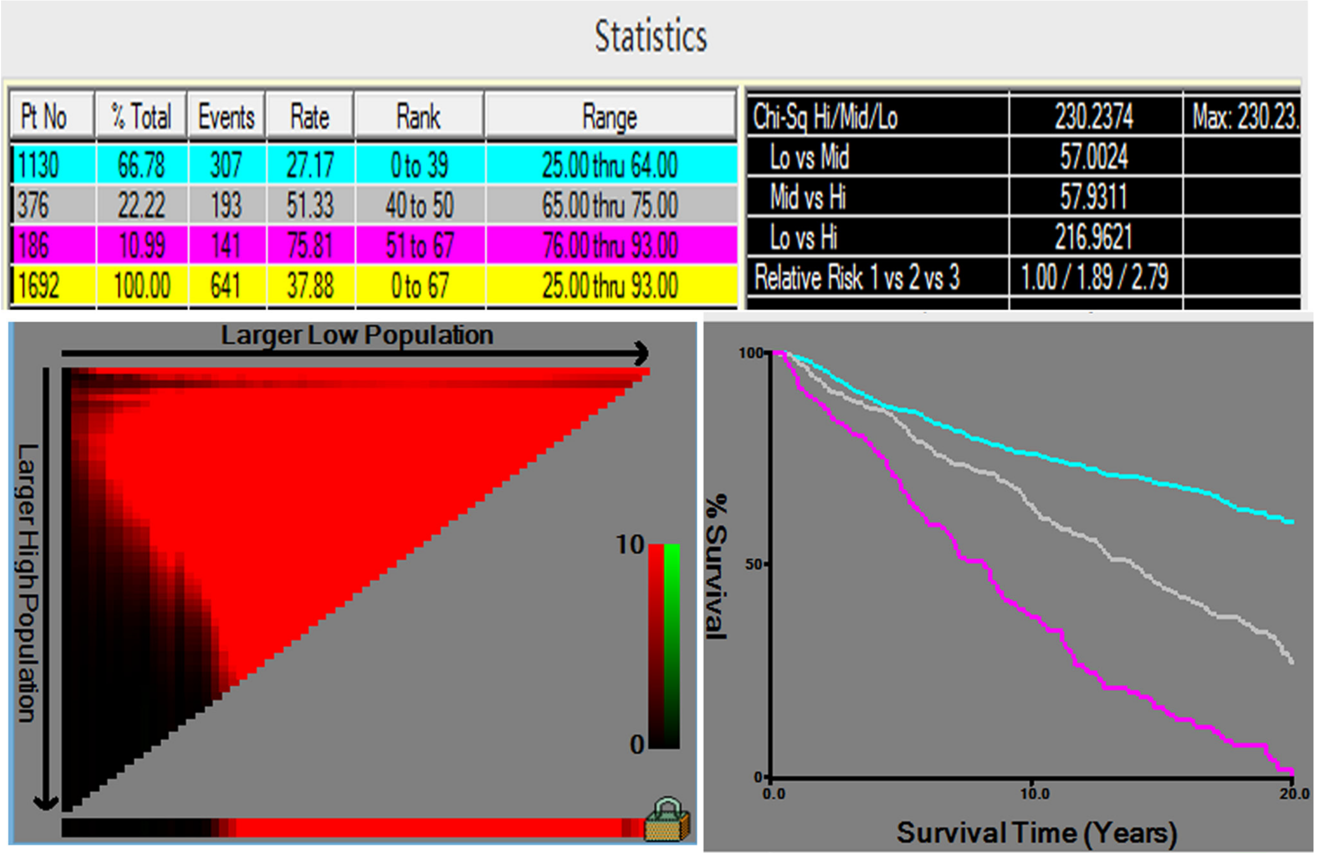

Supplementary Figure 1: The best cutoff points for age at diagnosed.
